# Supplementary figures and images for: Coalescent RNA-localizing and transcriptional activities of SAM68 modulate adhesion and subendothelial basement membrane assembly
Source: eLife. 2023 Aug 16;12:e85165. doi: 10.7554/eLife.85165 (PMC10431919; doi:10.7554/eLife.85165)

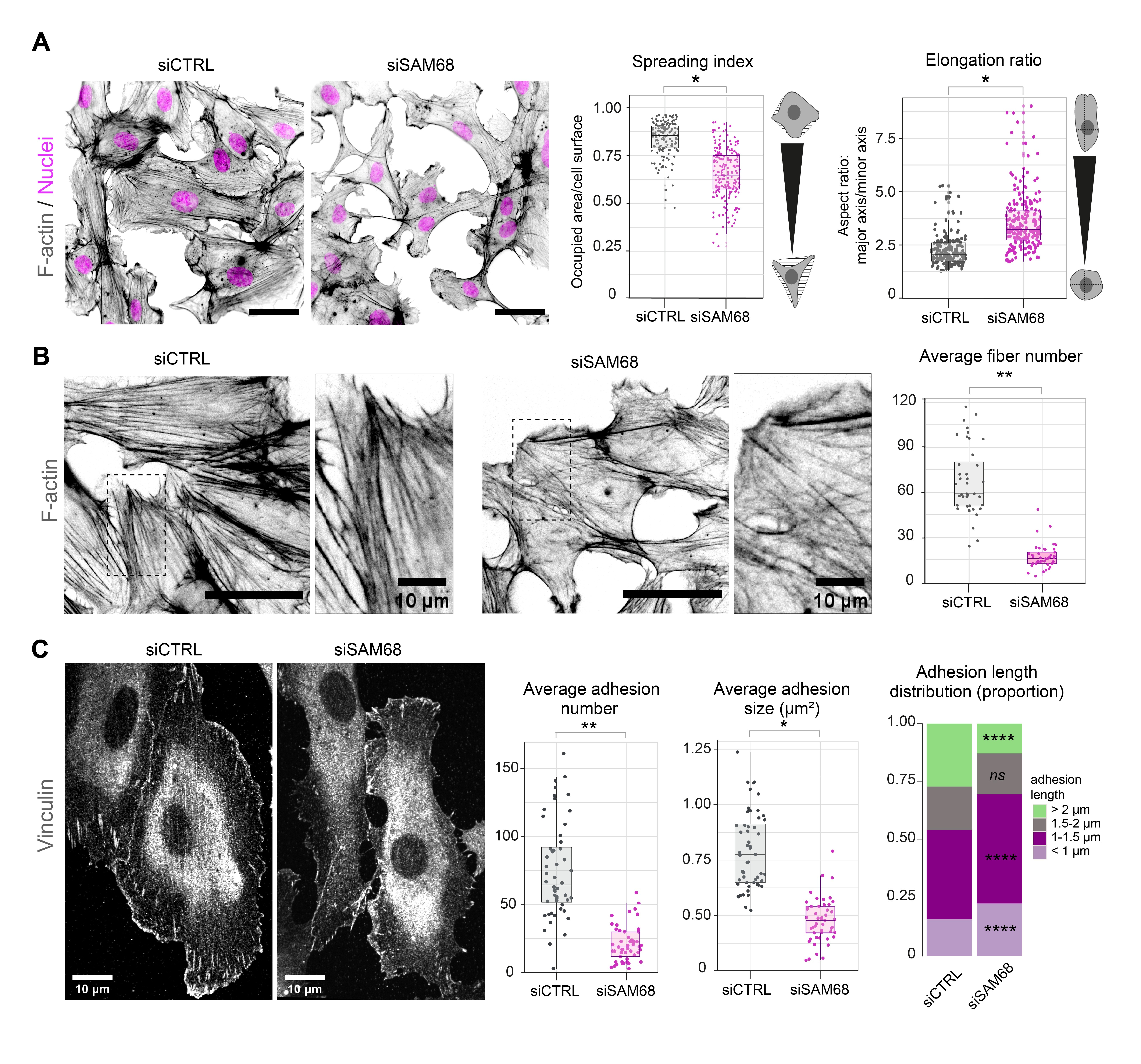

Supplement: Figure 1—source data 1. [file elife-85165-fig1-data1.zip › Rekad_et_al_Figure 1.jpg]

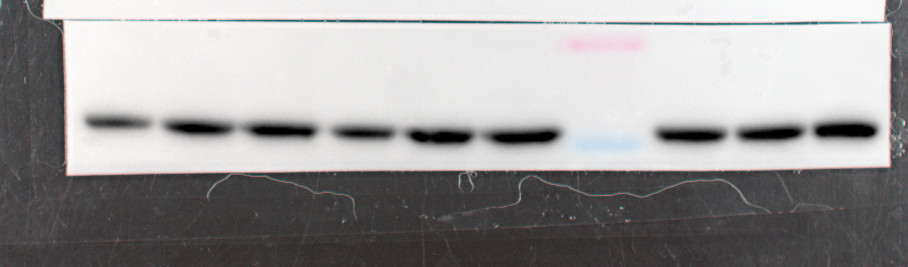

Supplement: Figure 1—figure supplement 1—source data 1. [file elife-85165-fig1-figsupp1-data1.zip › Fig 1 fig sup 1_HSP90.tif]

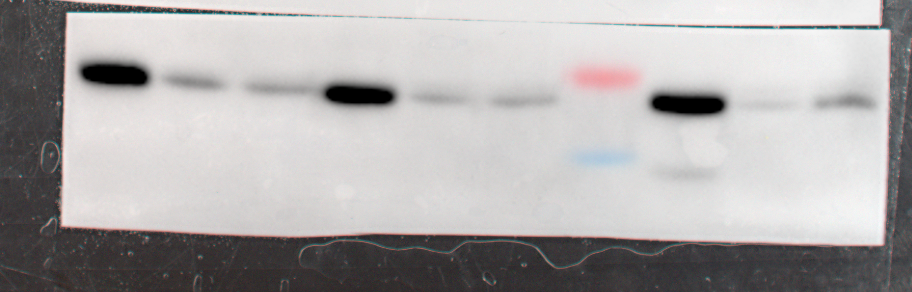

Supplement: Figure 1—figure supplement 1—source data 1. [file elife-85165-fig1-figsupp1-data1.zip › Fig 1 fig sup 1_SAM68.tif]

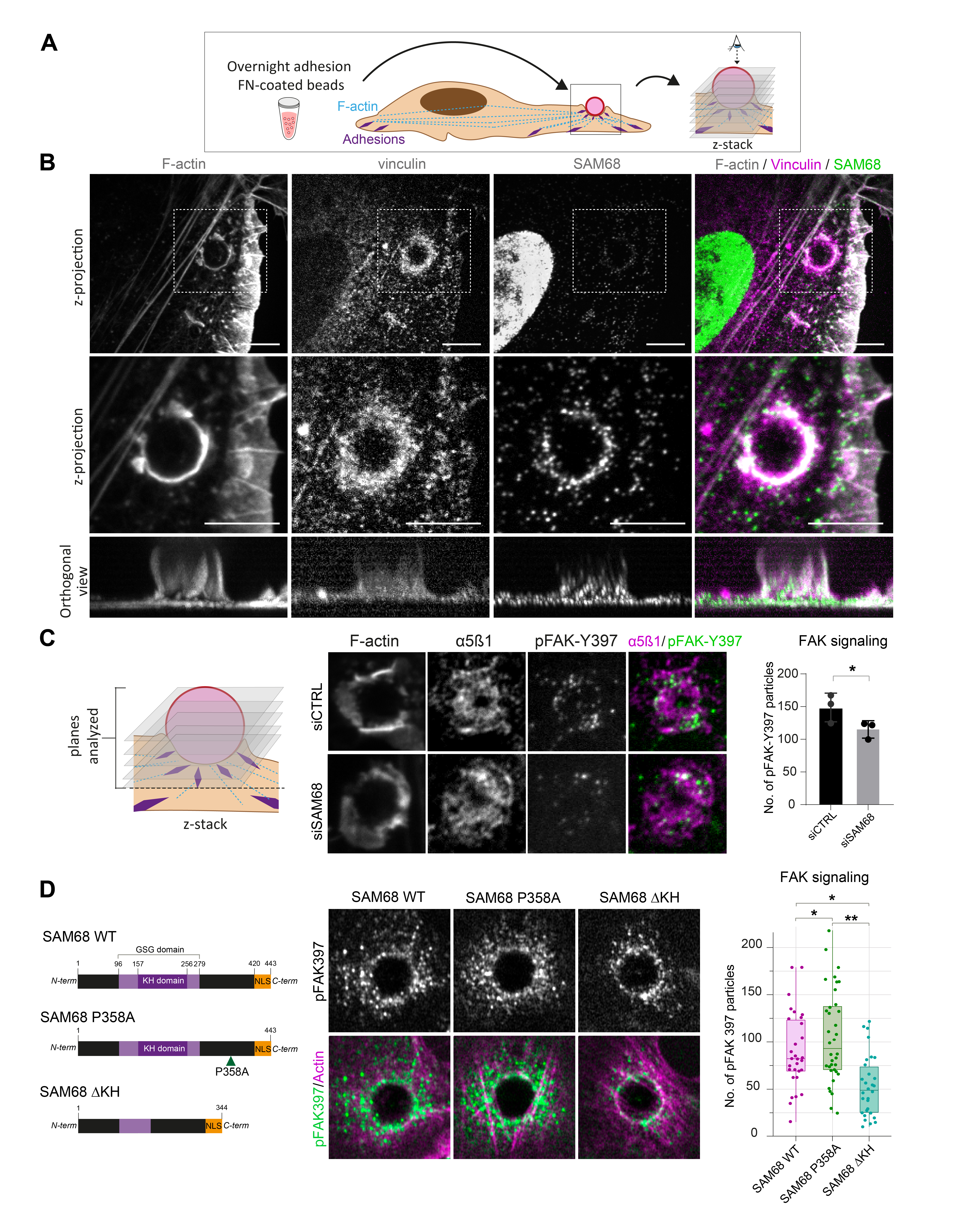

Supplement: Figure 3—source data 1. [file elife-85165-fig3-data1.zip › Rekad_et_al_Figure 3.jpg]

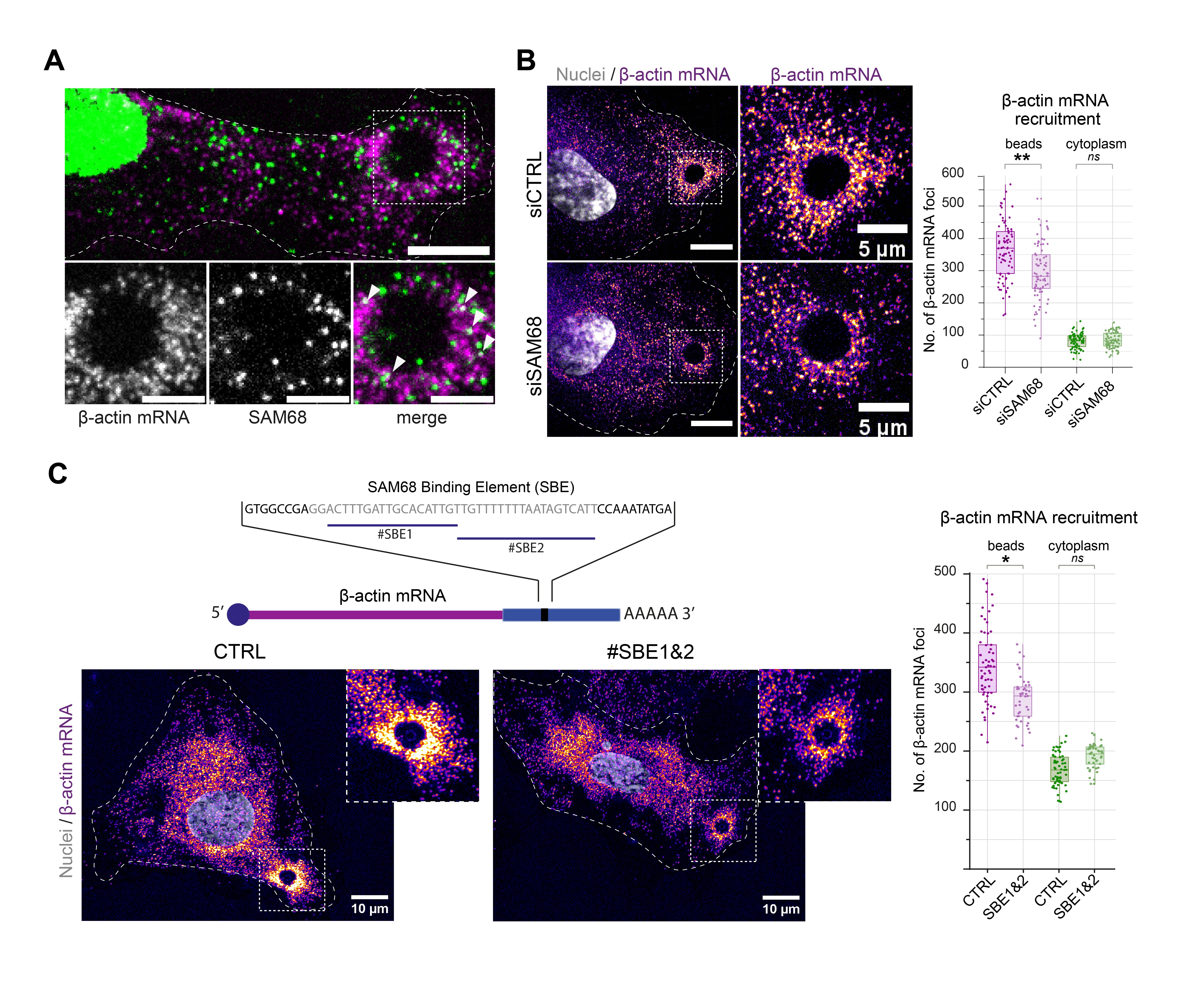

Supplement: Figure 4—source data 1. [file elife-85165-fig4-data1.zip › Rekad_et_al_Figure 4.jpg]

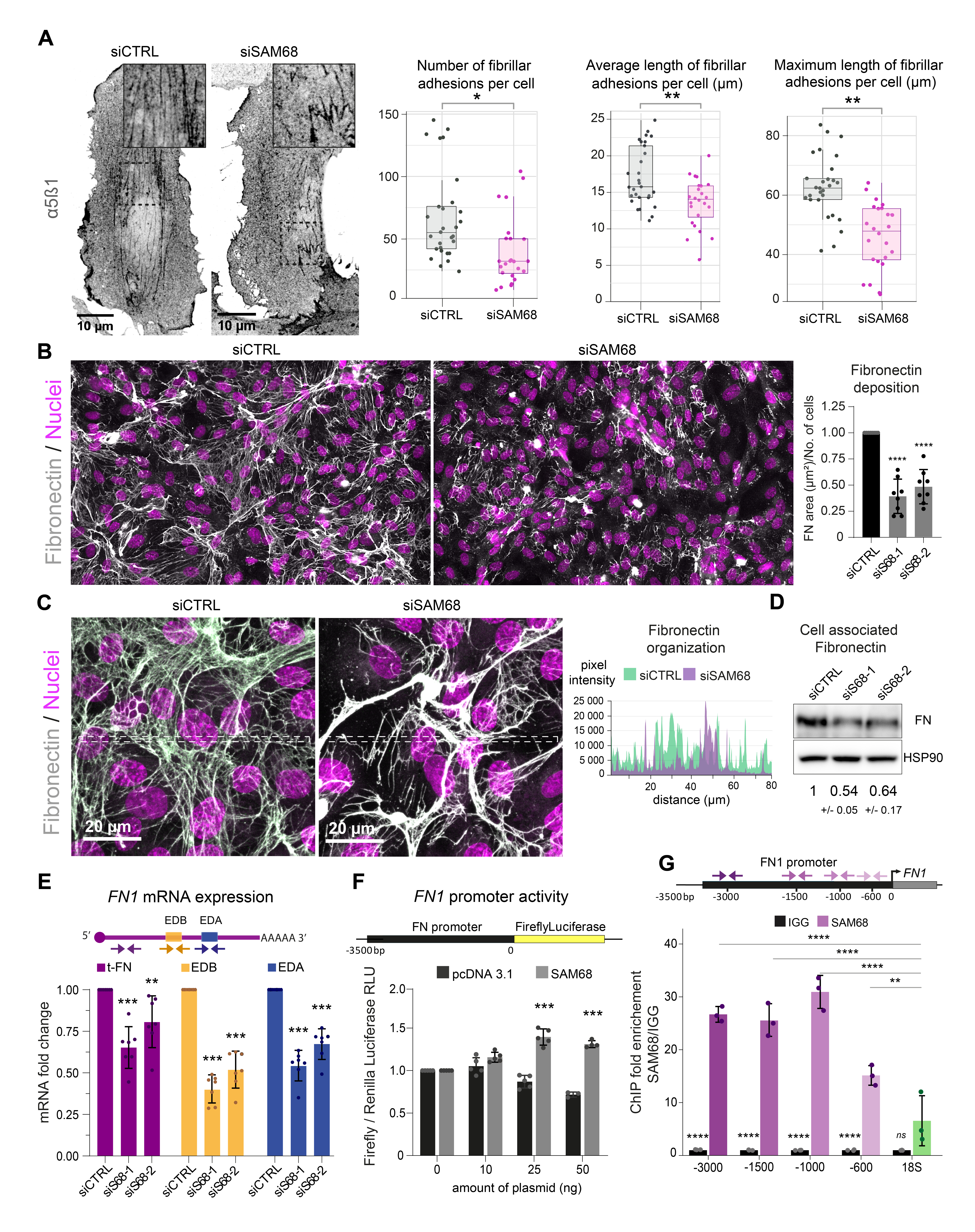

Supplement: Figure 5—source data 1. [file elife-85165-fig5-data1.zip › Rekad_et_al_Figure 5.jpg]

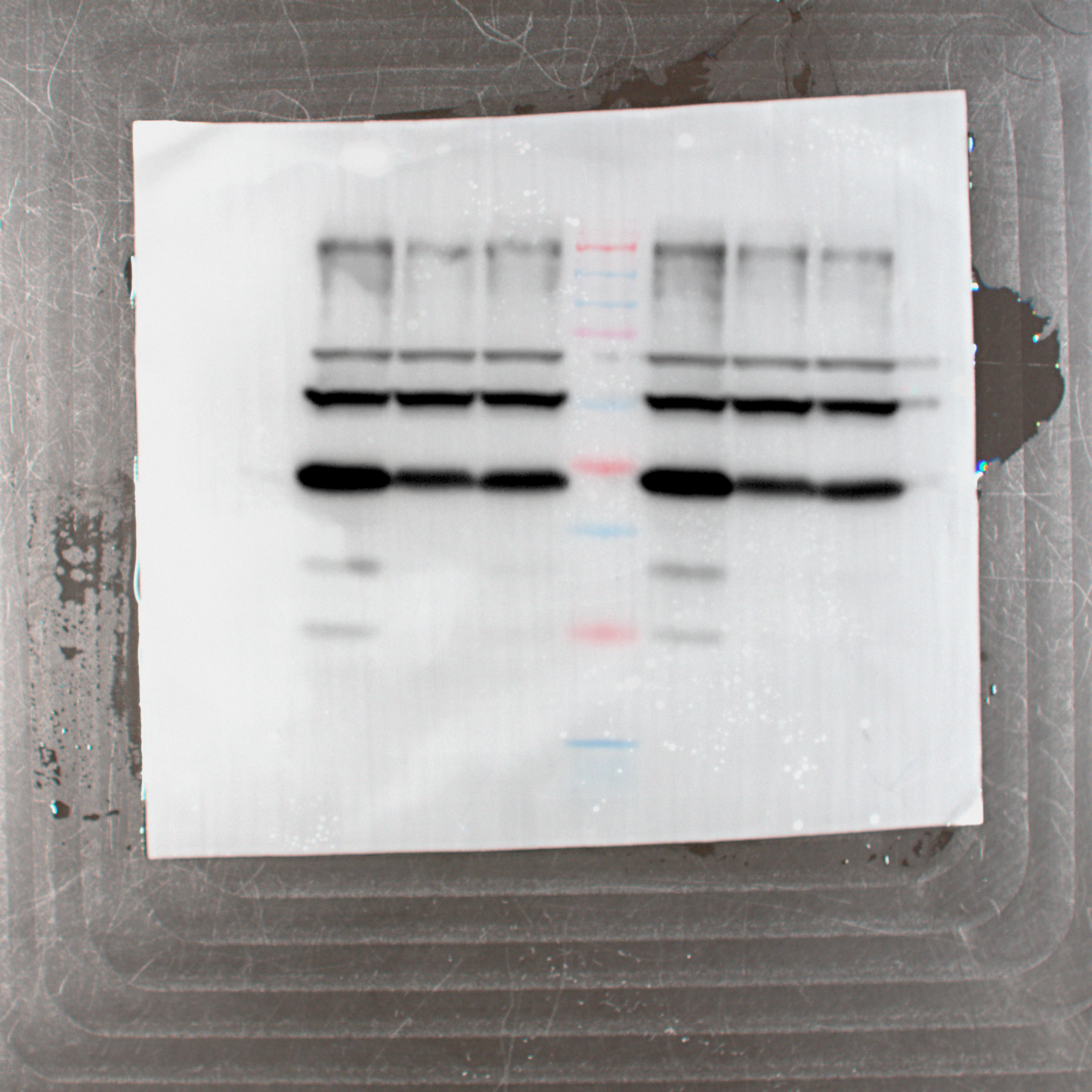

Supplement: Figure 5—source data 2. [file elife-85165-fig5-data2.zip › Figure 5D FN WB_uncut_FN_HSP90_SAM68.tif]

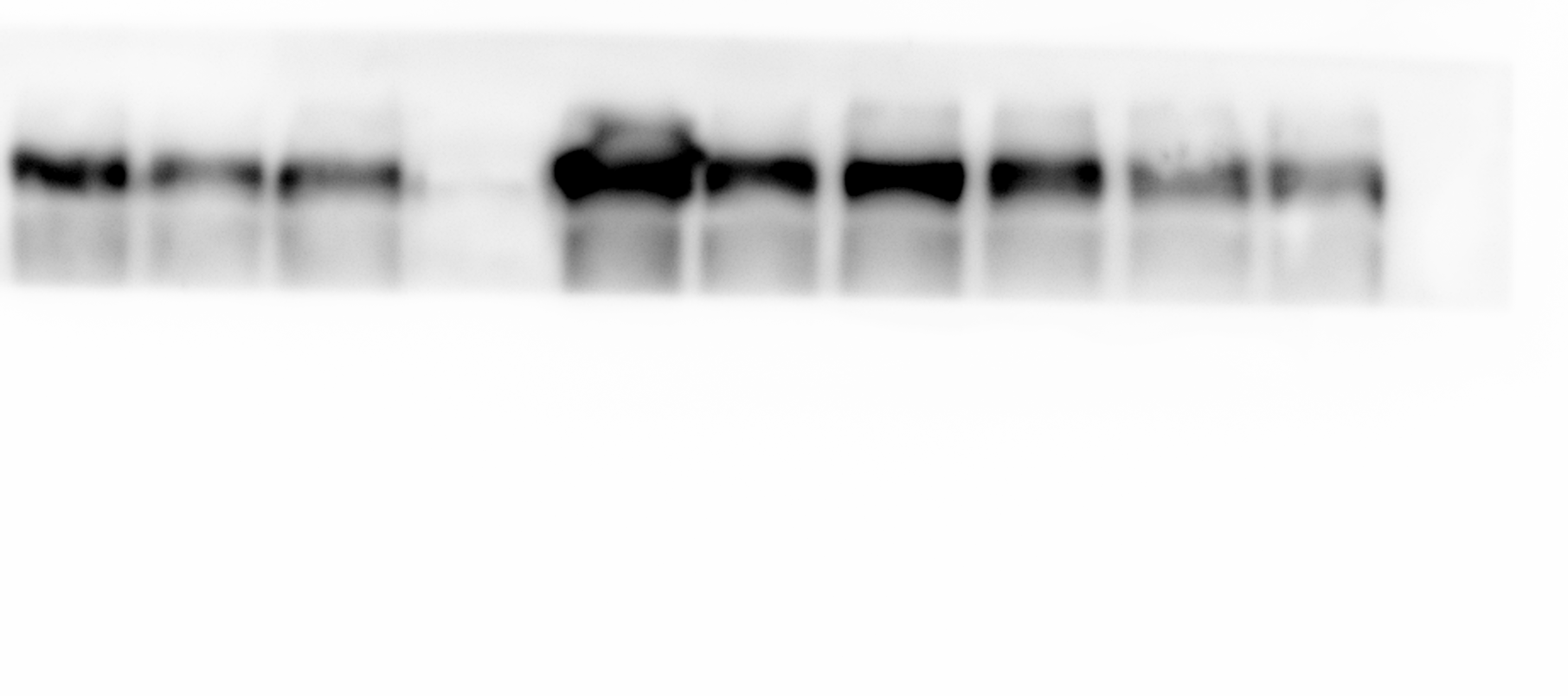

Supplement: Figure 5—source data 2. [file elife-85165-fig5-data2.zip › Figure 5D WB_cropped FN.tif]

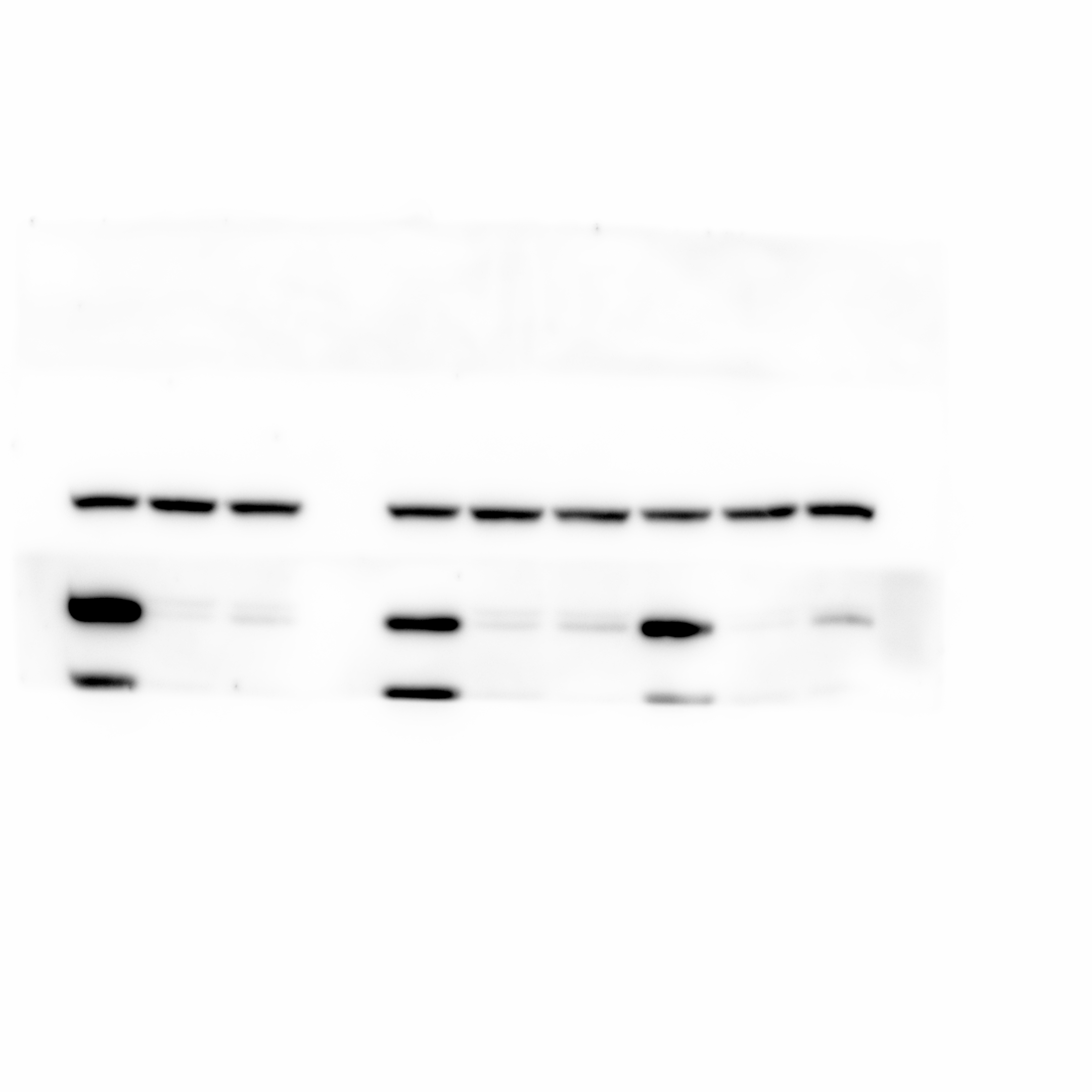

Supplement: Figure 5—source data 2. [file elife-85165-fig5-data2.zip › Figure 5D WB_cropped HSP90_SAM68.Tif]

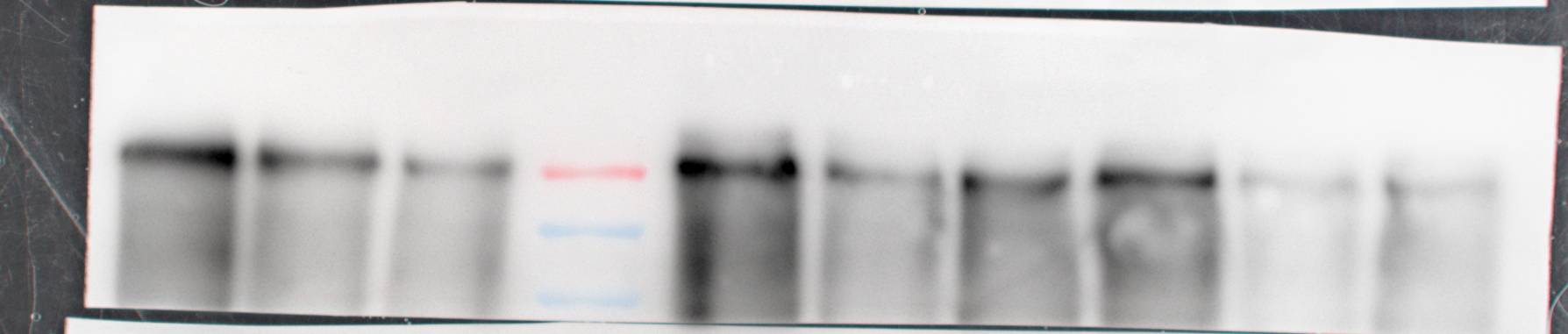

Supplement: Figure 5—figure supplement 1—source data 1. [file elife-85165-fig5-figsupp1-data1.zip › Fig 5 fig sup 1_ECMassoc_FN.tif]

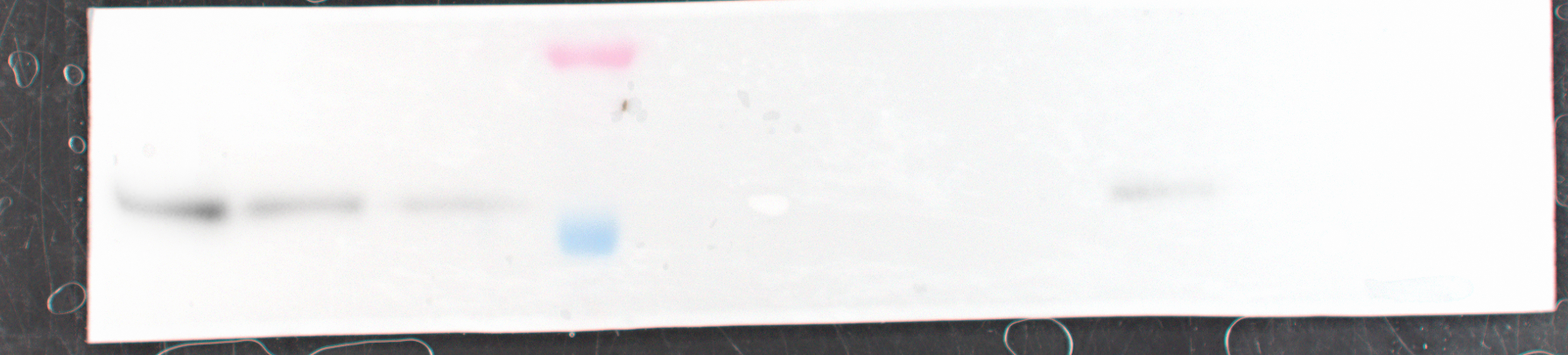

Supplement: Figure 5—figure supplement 1—source data 1. [file elife-85165-fig5-figsupp1-data1.zip › Fig 5 fig sup 1_ECMassoc_HSP90.tif]

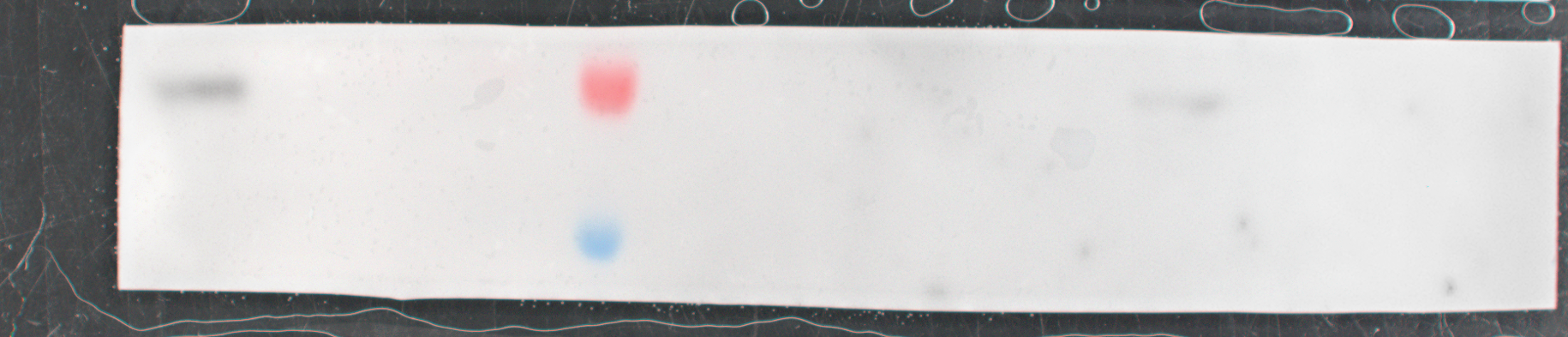

Supplement: Figure 5—figure supplement 1—source data 1. [file elife-85165-fig5-figsupp1-data1.zip › Fig 5 fig sup 1_ECMassoc_SAM68.tif]

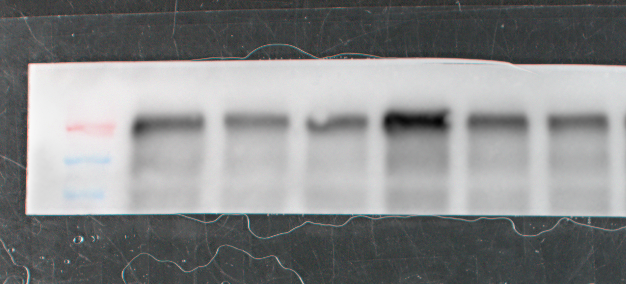

Supplement: Figure 5—figure supplement 1—source data 1. [file elife-85165-fig5-figsupp1-data1.zip › Fig 5 fig sup 1_Screted FN_FN.tif]

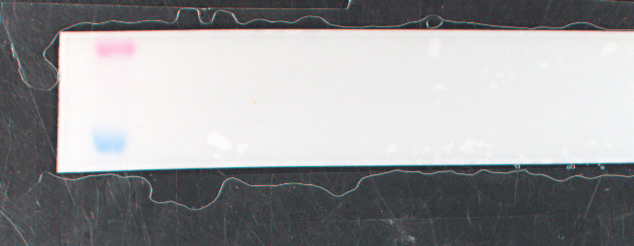

Supplement: Figure 5—figure supplement 1—source data 1. [file elife-85165-fig5-figsupp1-data1.zip › Fig 5 fig sup 1_Screted FN_HSP90.tif]

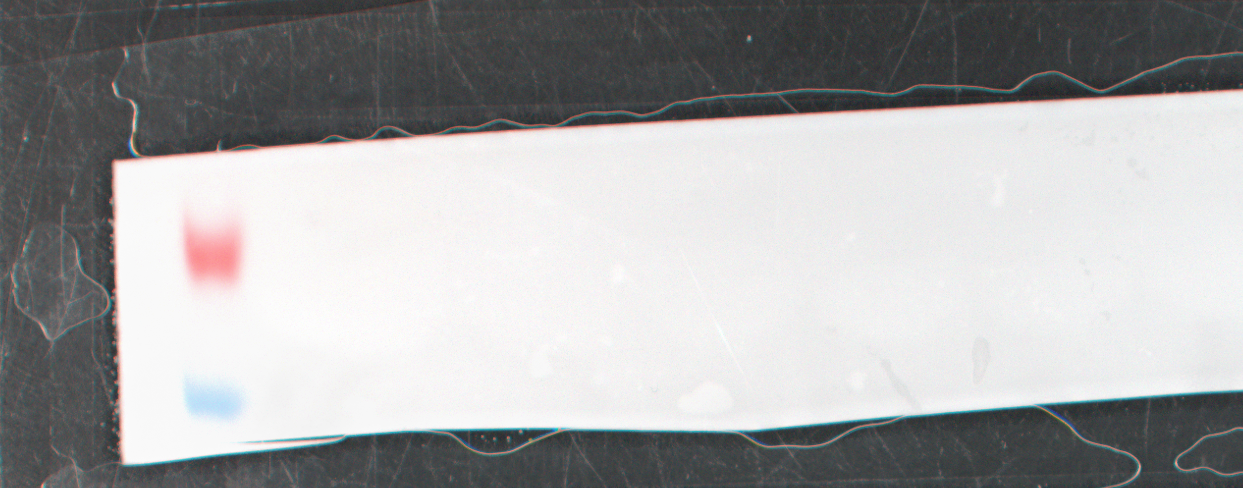

Supplement: Figure 5—figure supplement 1—source data 1. [file elife-85165-fig5-figsupp1-data1.zip › Fig 5 fig sup 1_Screted FN_SAM68.tif]

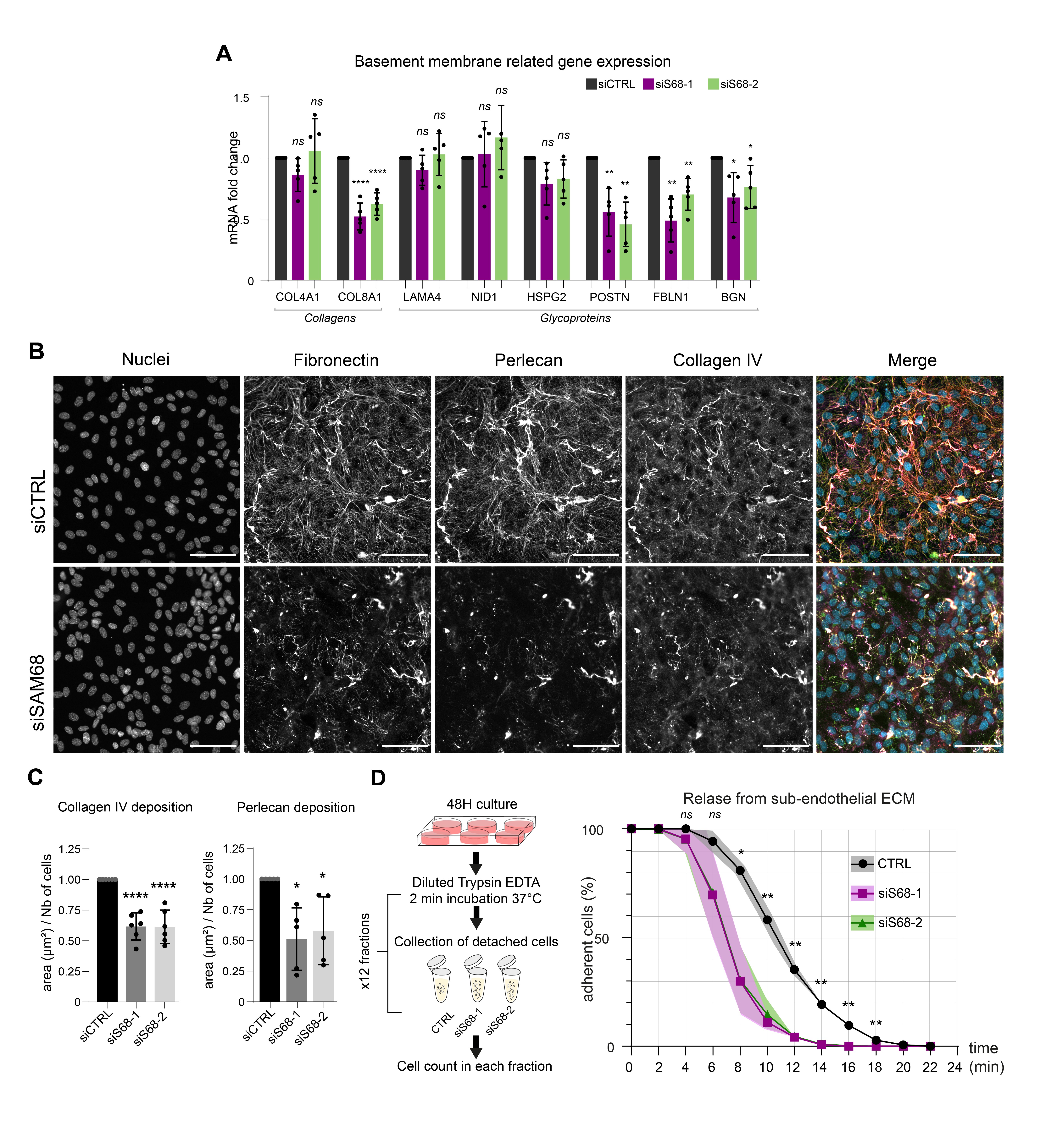

Supplement: Figure 6—source data 1. [file elife-85165-fig6-data1.zip › Rekad_et_al_Figure 6.jpg]

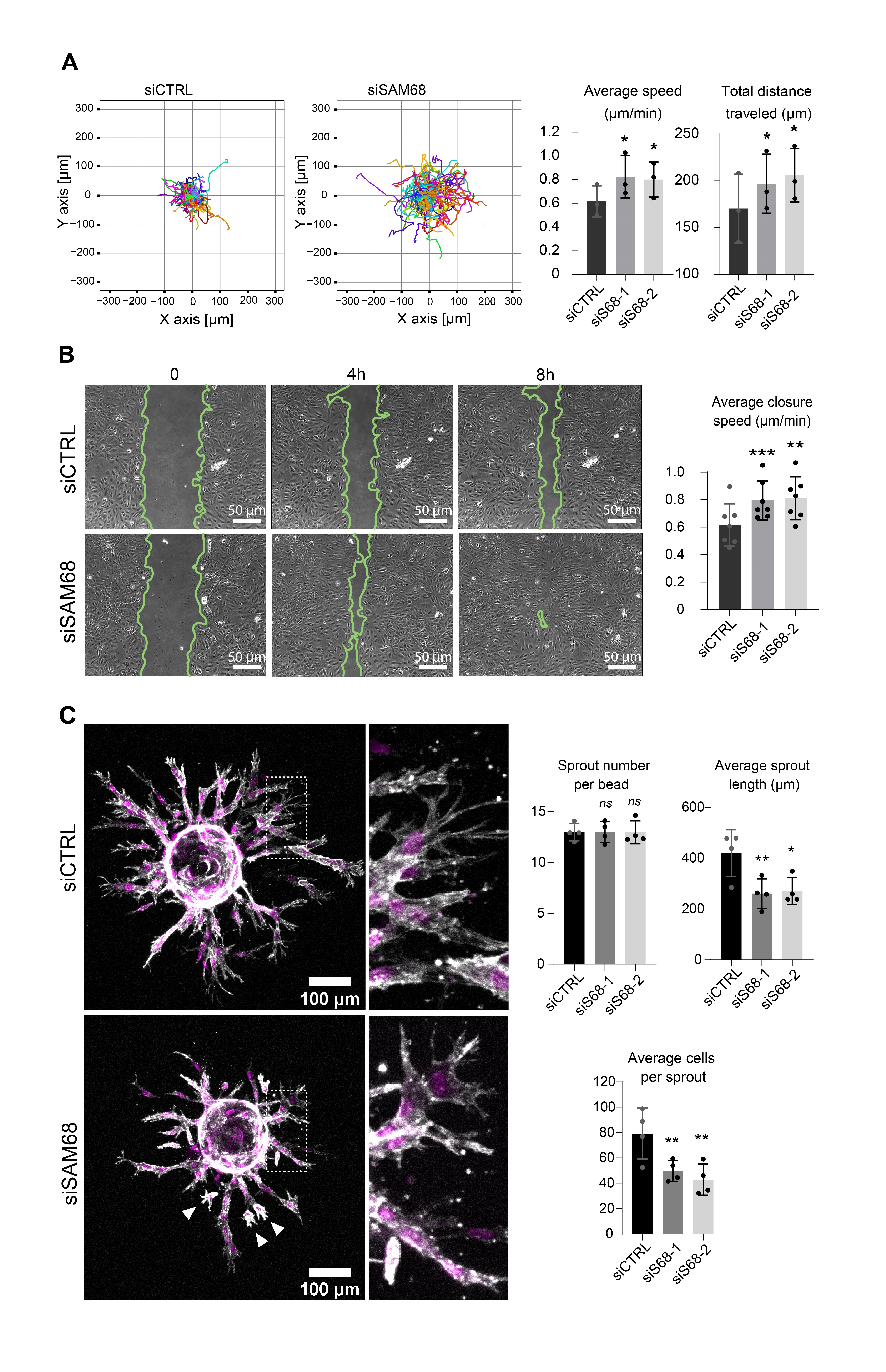

Supplement: Figure 7—source data 1. [file elife-85165-fig7-data1.zip › Rekad_et_al_Figure 7.jpg]
