# Supplementary material for: Coalescent RNA-localizing and transcriptional activities of SAM68 modulate adhesion and subendothelial basement membrane assembly
Source: eLife. 2023 Aug 16;12:e85165. doi: 10.7554/eLife.85165 (PMC10431919; doi:10.7554/eLife.85165)
Supplement: Figure 1—figure supplement 1—source data 1. [file elife-85165-fig1-figsupp1-data1.zip › Figure 1-figure supplement 1-source data 1.pptx]

## Slide 1
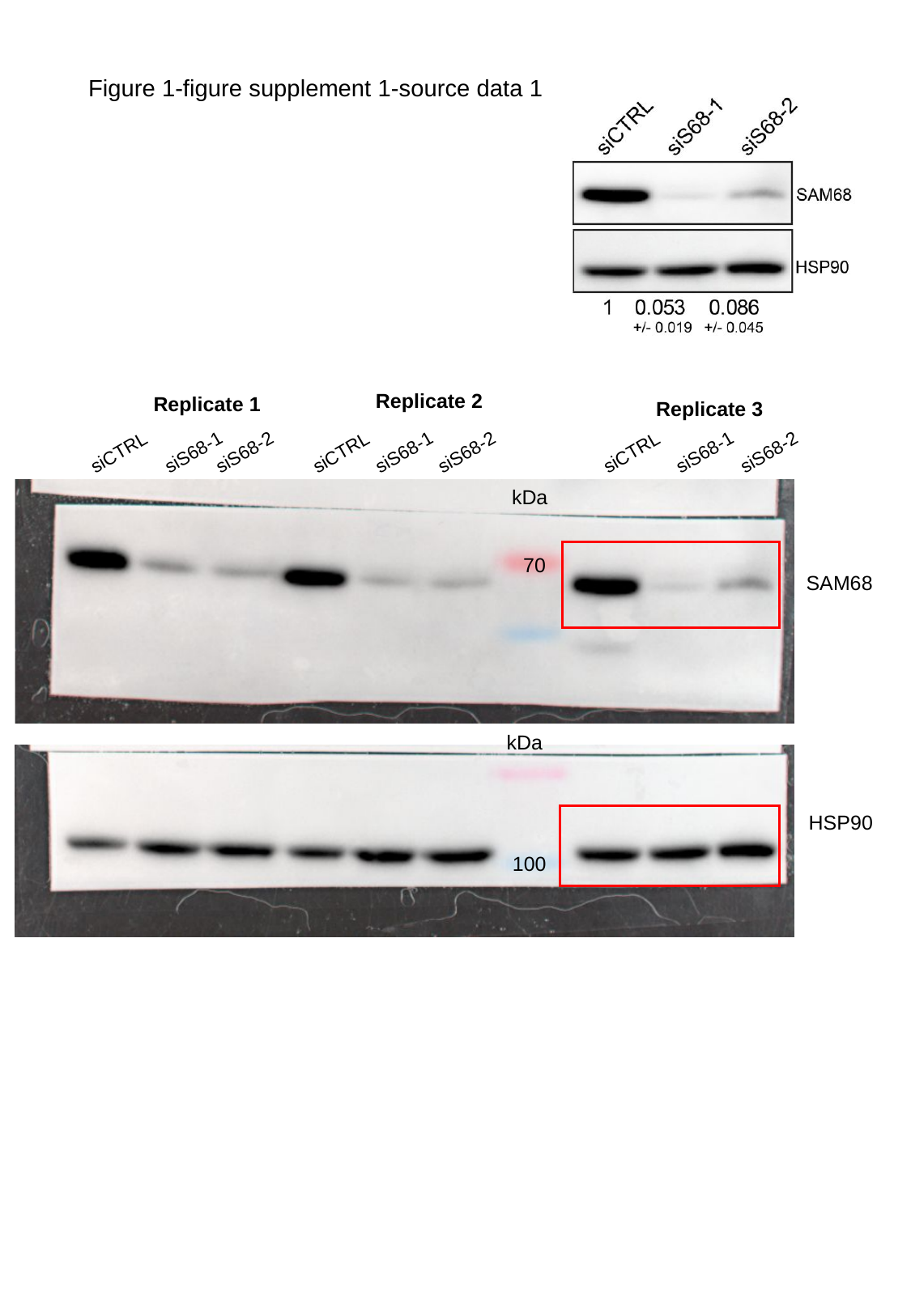

Figure 1-figure supplement 1-source data 1
Replicate 2
Replicate 1
Replicate 3
siS68-2
siS68-2
siS68-2
siS68-1
siS68-1
siS68-1
siCTRL
siCTRL
siCTRL
kDa
70
SAM68
kDa
HSP90
100
