# Supplementary material for: Coalescent RNA-localizing and transcriptional activities of SAM68 modulate adhesion and subendothelial basement membrane assembly
Source: eLife. 2023 Aug 16;12:e85165. doi: 10.7554/eLife.85165 (PMC10431919; doi:10.7554/eLife.85165)
Supplement: Figure 5—source data 2. [file elife-85165-fig5-data2.zip › Figure 5 source data 2.pptx]

## Slide 1
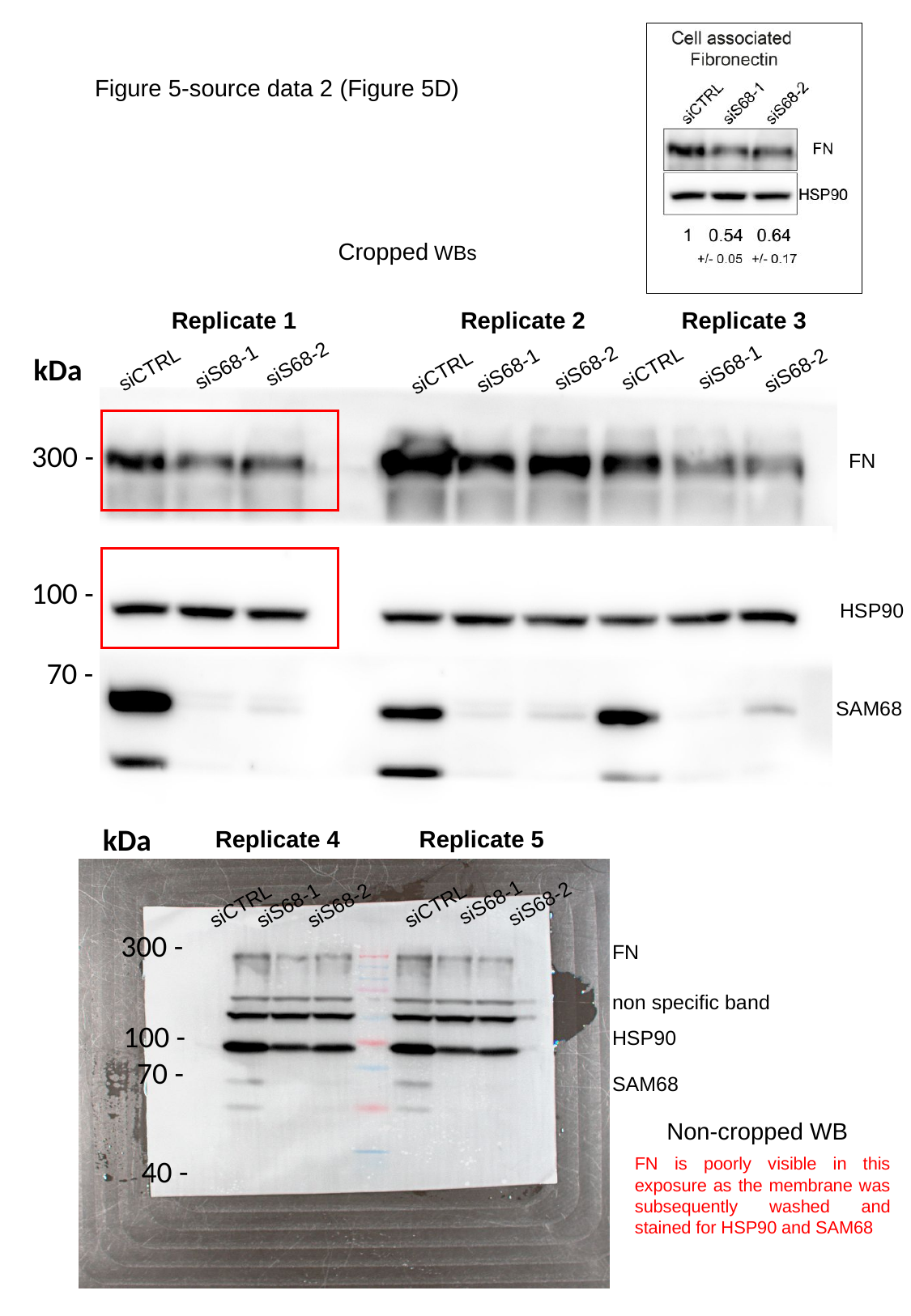

Figure 5-source data 2 (Figure 5D)
Cropped WBs
Replicate 2
Replicate 3
Replicate 1
siS68-2
siS68-1
siS68-2
siCTRL
siS68-1
siS68-1
siCTRL
siS68-2
siCTRL
FN
HSP90
SAM68
kDa
300 -
100 -
70 -
kDa
Replicate 4
Replicate 5
siS68-1
siS68-2
siS68-2
siS68-1
siCTRL
siCTRL
300 -
FN
non specific band
100 -
HSP90
70 -
SAM68
Non-cropped WB
FN is poorly visible in this exposure as the membrane was subsequently washed and stained for HSP90 and SAM68
40 -
