# Supplementary material for: Coalescent RNA-localizing and transcriptional activities of SAM68 modulate adhesion and subendothelial basement membrane assembly
Source: eLife. 2023 Aug 16;12:e85165. doi: 10.7554/eLife.85165 (PMC10431919; doi:10.7554/eLife.85165)
Supplement: Figure 5—figure supplement 1—source data 1. [file elife-85165-fig5-figsupp1-data1.zip › Figure 5_figure supplement 1_source data 1.pptx]

## Slide 1
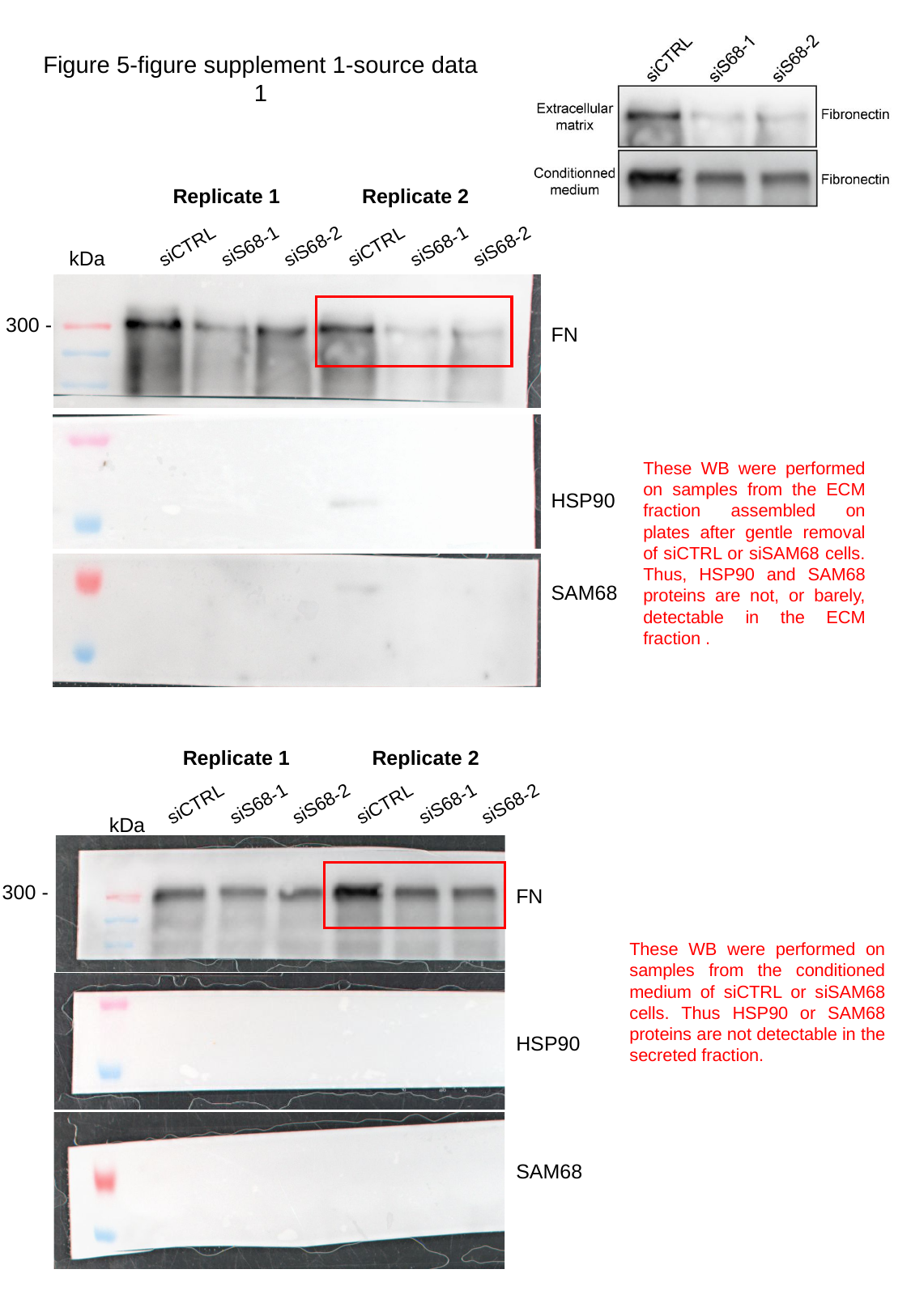

Figure 5-figure supplement 1-source data 1
Replicate 1
Replicate 2
siS68-2
siS68-2
siS68-1
siS68-1
siCTRL
siCTRL
kDa
300 -
FN
These WB were performed on samples from the ECM fraction assembled on plates after gentle removal of siCTRL or siSAM68 cells. Thus, HSP90 and SAM68 proteins are not, or barely, detectable in the ECM fraction .
HSP90
SAM68
Replicate 1
Replicate 2
siS68-2
siS68-2
siS68-1
siS68-1
siCTRL
siCTRL
kDa
300 -
FN
These WB were performed on samples from the conditioned medium of siCTRL or siSAM68 cells. Thus HSP90 or SAM68 proteins are not detectable in the secreted fraction.
HSP90
SAM68
